# The potential contribution of dietary factors to breast cancer prevention

**DOI:** 10.1097/CEJ.0000000000000406

**Published:** 2017-08-02

**Authors:** Niva Shapira

**Affiliations:** Department of Nutrition, School of Health Professions, Ashkelon Academic College, Ashkelon, Israel

**Keywords:** antioxidants, breast cancer, DNA adducts, estrogen, metabolic syndrome, nutritional prevention sex nutrition, obesity, plant-based diet

## Abstract

Breast cancer (BC), the leading cancer in women, is increasing in prevalence worldwide, concurrent with western metabolic epidemics, that is, obesity, metabolic syndrome, and diabetes, and shares major risk factors with these diseases. The corresponding potential for nutritional contributions toward BC prevention is reviewed and related to critical stages in the life cycle and their implications for carcinogenic and pathometabolic trajectories. BC initiation potentially involves diet-related pro-oxidative, inflammatory, and procarcinogenic processes, that interact through combined lipid/fatty acid peroxidation, estrogen metabolism, and related DNA-adduct/depurination/mutation formation. The pathometabolic trajectory is affected by high estrogen, insulin, and growth factor cascades and resultant accelerated proliferation/progression. Anthropometric risk factors – high birth weight, adult tallness, adiposity/BMI, and weight gain – are often reflective of these trends. A sex-based nutritional approach targets women’s specific risk in western obesogenic environments, associated with increasing fatness, estrogen metabolism, *n*-6 : *n*-3 polyunsaturated fatty acid ratio, and *n*-6 polyunsaturated fatty acid conversion to proinflammatory/carcinogenic eicosanoids, and effects of timing of life events, for example, ages at menarche, full-term pregnancy, and menopause. Recent large-scale studies have confirmed the effectiveness of the evidence-based recommendations against BC risk, emphasizing low-energy density diets, highly nutritious plant-based regimes, physical activity, and body/abdominal adiposity management. Better understanding of dietary inter-relationships with BC, as applied to food intake, selection, combination, and processing/preparation, and recommended patterns, for example, Mediterranean, DASH, plant-based, low energy density, and low glycemic load, with high nutrient/phytonutrient density, would increase public motivation and authoritative support for early/timely prevention, optimally merging with other dietary/health goals, for lifelong BC prevention.

## Background

Breast cancer (BC) is the most common cancer among women worldwide, with increasing prevalence, particularly the postmenopausal type, and in areas where the incidence had previously been low, such as Japan, China, and southern and eastern Europe (WCRF, AICR, 2007). This epidemiological pattern, which follows those of other western epidemics, and shares similar risk factors – obesity, type 2 diabetes mellitus (T2DM), and cardiovascular disease – strongly suggests that it is part of the pathometabolic prevalence that is closely associated with western lifestyle patterns, and thus may support a nutritional approach to BC prevention.

Among BC cases, only 5–10% were because of genetic defects, with 90–95% attributable to environmental and lifestyle factors – diet and obesity contributing ∼30–35 and 10–20%, respectively – providing major opportunities for nutritional prevention (Anand *et al.*, 2008). Among the 5–10% of genetically based BC cases, many were caused by inherited mutations in either the *BRCA1* or the *BRCA2* genes (Anderson and Badzioch, 1993; Blackwood and Weber, 1998), which, according to the number of cohorts, showed a marked increase in penetration over the recent decades, possibly reflecting increased western reproductive and lifestyle risk factors, including western diet, over-fatness, smoking, and low physical activity (Friebel *et al.*, 2014; Pettapiece-Phillips *et al.*, 2015).

### Critical periods for breast cancer risk and prevention

Early critical periods – before attaining BC protection by cell differentiation through a full-term pregnancy-induced ‘molecular signature’ (Barton *et al.*, 2014) – are associated with endocrine/metabolic predisposition. Late menarche, early pregnancy/childbearing, and early menopause – all related to reduced number of menstrual cycles, exposure to estrogen, and periods of accelerated cell proliferation, whereas high birth weight (WCRF, AICR, 2007), early menarche, late menopause, and/or no or late (age>30 years) childbearing have been shown to increase BC risk (MacMahon, 1993; McPherson *et al.*, 2000; Barton *et al.*, 2014), thus offering opportunities for early nutritional protection (de Boo and Harding, 2006; Swanson *et al.*, 2009).

Whereas overconsumption has been shown to lead to large birth weight (WCRF, AICR, 2007), to early puberty, telarche, and menarche, and to delayed menopause, lower BMI has beneficially delayed puberty and advances the age of menopause (WCRF, AICR, 2007), suggesting that each of these life stages potentially becomes a ‘window of risk’ – particularly with the western obesogenic diet – or ‘a window of opportunity’ – for BC prevention by nutritional/lifestyle management (de Waard and Trichopoulos, 1988; Stoll, 1998; Berkey *et al.*, 1999; Shapira, 2001; Colditz *et al.*, 2014).

### Endometabolic mechanisms related to breast cancer

Various mechanisms by which diet and lifestyle may promote BC have been reviewed previously (Kaaks, 1996; Vainio and Bianchini, 2002; Lorincz and Sukumar, 2006), suggesting that a sedentary lifestyle, overweight, and a fat-rich diet are associated with insulin resistance and increased androgenic activity, whereas physical activity improves insulin sensitivity and decreases testosterone and insulin-like growth factor 1 (IGF-1) levels. Insulin stimulates the synthesis of androgens in the ovary and the expression of growth hormone receptors, and inhibits liver production of sex hormone-binding globulin and IGF binding proteins 1 and 2, thus increasing the bioavailability of both sex hormones and IGF-1. Postmenopausal overweight is associated with increased peripheral conversion of androgens to estrogens, decreased sex hormone-binding globulin, and increased insulin levels, especially among obese smokers and drinkers (Endogenous Hormones Breast Cancer Collaborative Group *et al.*, 2011), and alcohol intake further increases the synthesis of androgens and estrogens (Rinaldi *et al.*, 2006). Across ages, control of adiposity and dysmetabolism can reduce the risk of aggressive BC subtypes and improve the prognosis (Agresti *et al.*, 2016).

Beyond postmenopausal estrogen sources from adipose tissue aromatization, 27-hydroxycholesterol (27HC) was recently shown to exert an estrogen-like effect, acting as a local endogenous selective estrogen receptor (ER) modulator, which is potentially reduced by anticholesterol drugs and phytonutrients (McDonnell *et al.*, 2014a).

Estrogen’s association with BC operates by both hormonal ER-mediated stimulation of breast cell proliferation, with enhanced chances of mutations, and estradiol’s genotoxic metabolites that generate oxygen free radicals and alterations, initiating DNA mutagenic processes (Cavalieri and Rogan, 2014); both mechanisms are reduced by a variety of estrogen inhibitors (Santen *et al.*, 2015). High urine DNA adducts in at-risk or active BC cases indicate a critical role for adduct formation in BC initiation and potential use of antioxidants capable of blocking estrogen-DNA-adduct formation and depurination, for example, *N*-acetylcysteine and resveratrol, which have shown inhibitory potential *in vitro* and *in vivo* (Cavalieri and Rogan, 2010).

### Sex-based nutrition and female specificity

Women’s differential body fat accumulation and distribution, which is increasingly manifested during puberty/adolescence, shows lower abdominal and visceral fat accumulation versus a tendency toward higher gluteal and subcutaneous accumulation than men, and body fat percentage higher than that of men, even with equal BMI; moreover, their lower fat loss on weight-reduction diets, better response to high-protein versus high-carbohydrate diets, higher risks with sedentariness versus greater benefits with exercise, and tendency toward delayed onset of central obesity, metabolic syndrome (MetS), T2DM, cardiovascular disease, and certain cancers – until menopause but accelerated thereafter – together reflect women’s differential metabolic and chronological life cycle patterns (Shapira, 2013).

The postmenopausal state causes fat redistribution to an androgenic pattern, with increasing abdominal adiposity and related metabolic risks, including decreased insulin and leptin sensitivities, and changes in glucose and lipid metabolism, resulting in reduced energy expenditure and increased weight gain and obesity, potentially contributing toward the development of BC. This is despite reduced ovarian estrogen secretion, while increasing localized inflammation and estrogen production in breast tissue, and growth factor secretion (Boonyaratanakornkit and Pateetin, 2015).

Together, the above suggests women’s need for specific metabolic and chronological perspectives for prevention/intervention, especially against BC, which closely represents the female life cycle pattern as related to endocrine-metabolic and diet-dependent risks (Shapira, 2013).

### Recommended lifestyle changes for breast cancer prevention

#### Lifestyle factors

On the basis of the available evidence (WCRF, AICR, 2007, 2010; Eccles *et al.*, 2013), recommendations from the World Cancer Research Fund and the American Institute for Cancer Research on diet, physical activity, and weight management include the following: (i) maintain adequate body weight; (ii) be physically active; (iii) limit the intake of high-energy density (ED) foods; (iv) eat mostly plant foods; (v) limit the intake of animal foods; (vi) limit alcohol intake; (vii) limit salt and salt-preserved food intake; and (viii) meet nutritional needs through diet; and special recommendations (S1) breastfeed infants exclusively up to 6 months and (S2) after cancer treatment–follow the recommendations for BC prevention. Associations between each of these recommendations and BC risk (Romaguera *et al.*, 2012; Hastert *et al.*, 2013; Catsburg *et al.*, 2014; Thomson *et al.*, 2014), across tumor subtypes, and considering hormonal receptors and the human epidermal growth factor receptor 2 (*HER2*) status (Castello *et al.*, 2015), have yielded encouraging results. Accordingly, adherence to only three versus six or more recommendations increased the risk [odds ratio (OR)≈3.00–4.00] for premenopausal and postmenopausal women, respectively.

For postmenopausal women, the three leading recommendations were to eat a plant-based diet, limit high-ED foods, and maintain adequate body weight. Both low-ED and plant-based foods with high fiber and water contents are expected to be more satiating and contribute toward body weight management. Limiting intake of animal foods yielded only a minimal advantage, despite previous recommendations. High sugar intake, particularly in sugar-sweetened beverages (SSB), has shown a direct association with obesity, MetS, and diabetes (Malik *et al.*, 2010; Barrio-Lopez *et al.*, 2013). Alcohol was associated progressively with increased risk (≈35%) for both postmenopausal and premenopausal women. In premenopausal women, ‘not limiting high-ED foods’ increased almost two-fold the risk of BC, and increased sugar intake predicted earlier age at menarche (Carwile *et al.*, 2015). Correspondingly, lower rates of BC risk (by 16–60%) were found with increased adherence to the World Cancer Research Fund/American Institute for Cancer Research guidelines – which are linked to reduced body fatness and alcohol intake (Hastert *et al.*, 2013; Thomson *et al.*, 2014; Makarem *et al.*, 2015; McKenzie *et al.*, 2015; Kohler *et al.*, 2016) – and with ‘adherence to whole grain products’ and ‘reduced meat and alcohol’ (Catsburg *et al.*, 2014). These effects were shown for both ER+ and ER− BCs (Hastert *et al.*, 2013; McKenzie *et al.*, 2015) and with increased penetration of *BRCA2* (1920–2000) and BC prevalence in the general population, together suggesting that all BC types may potentially benefit from the above preventive measures (Tryggvadottir *et al.*, 2006).

#### Dietary patterns

*Whole food plant-based and low-ED diet*: A whole food plant-based diet, which is also tends to be low ED, potentially supports body weight management, and is innately high in micronutrients, including vitamins, minerals, fiber, and phytonutrients, like antioxidants from vegetables, fruits, and whole grains and beans (Assi *et al.*, 2015), which are necessary for enabling proper metabolic patterns. Some of their bioactive compounds – carotenoids, polyphenols, and isothiocyanates – have documented cancer-preventive activity, observed by linear reduction (OR=0.66) of BC with the ‘salad vegetable’ pattern in the ORDET study (Sieri *et al.*, 2004), together explaining how high intake of vegetables and fruits with olive oil (Sieri *et al.*, 2004) has protective potential against BC.

### Traditional diets

Some balanced ethnic patterns, such as the Mediterranean diet, were shown to be more easily and successfully translated and applied than the analytical recommendations on the basis of dietary composition, and can thus highly contribute toward dietary prevention of western diseases – including against BC.

#### Mediterranean diet

The Mediterranean diet pattern is fundamentally plant rich and low ED, providing high amounts of antioxidant flavonoids, carotenoids, and vitamins, plus phytoestrogens, fiber, folate, and a favorable fatty acids (*n*-3 :  *n*-6) profile (Chlebowski *et al.*, 1986; Berclaz *et al.*, 2004). The DIANA interventional trials showed that Mediterranean dietary principles can reduce body weight, improved fat distribution, reduce insulin levels, and MetS factors, as well as the bioavailability of sex hormones and growth factors (Berrino *et al.*, 2001; Kaaks *et al.*, 2003; Berrino *et al.*, 2005). In a principal component analysis on vegetables, fruit, fish, and legumes, each was associated independently with decreased adjusted risk (OR=0.67) (Demetriou *et al.*, 2012).

In the recent PREDIMED randomized clinical intervention trial, the multivariable-adjusted hazard ratios versus the control group were lower – 0.32 for the Mediterranean diet with extra-virgin olive oil and 0.59 for the Mediterranean diet with nuts group (Toledo *et al.*, 2015). Even where high-Mediterranean diet adherence did not protect against BC following removal of alcohol intake from the diet score (Schwingshackl and Hoffmann, 2014), it did reduce mortality from other causes (Kim *et al.*, 2011; Izano *et al.*, 2013; de Lorgeril and Salen, 2014).

#### Okinawan diet

The Okinawan diet, similar to the Mediterranean pattern – very low ED, glycemic load (GL), and fat, while high in fiber, micronutrients, phytochemicals, prebiotic/probiotic, and *n*-3 polyunsaturated fatty acids (PUFA) – from whole grains, beans, fruits, vegetables, fermented products, and marine foods, eaten fresh/raw or lightly cooked, with limited red meat and *n*-6 PUFA – has yielded one of the longest-living populations in the world (Tamaki *et al.*, 2015), with many of the traditional foods, herbs, and spices consumed on a regular basis considered ‘functional foods’ (Willcox *et al.*, 2009). However, local BC mortality has increased concurrently with reduced dietary adherence (Tamaki *et al.*, 2015) and body weight gain of Okinawan women (Tamaki *et al.*, 2014), confirming the effectiveness of the traditional diet and corroborating its structural similarity to the Mediterranean diet (Willcox *et al.*, 2009; Demetriou *et al.*, 2012), thus highly relevant for BC prevention in other populations as well.

### Dietary factors

Beyond general diet characteristics, individual dietary factors can also play an important role either in the development or in the prevention of BC (Rossi *et al.*, 2014), that is, whereas red meat (especially ‘well-done’), fat, sugars, and high GL are among the risk factors, whole foods from plant-based and marine sources with high nutrient and phytonutrient density make protective contributions. Some nutrients were shown to exert specific protective effects against the development of BC that are inherently insufficient in the diet, for example, vitamin D (Rossi *et al.*, 2014) and *n*-3 PUFA (Rose, 1993; Simonsen *et al.*, 1998).

#### Energy density

Limiting the intake of high-ED foods is a well-known strategy to attain higher satiety by lower caloric intake (Ello-Martin *et al.*, 2005). This is because portion size is more closely associated with satiety than its caloric content. Increasing the amounts of fruits and vegetables, starting the meal with a soup or salad and/or with a low-calorie preload, and/or low-fat/low-carbohydrate diets have all been shown to contribute successfully toward reduced caloric intake and better body weight management (Rolls, 2012), and were recently suggested as a leading BC-prevention strategy (WCRF, AICR, 2010; Eccles *et al.*, 2013).

#### Fat

Despite the previous assumption that dietary fat affects BC, similar to other western diseases – possibly through its contribution to passive overconsumption and resultant overweight and related pathometabolic effects – there is only limited evidence overall suggesting effects on postmenopausal BC (WCRF, AICR, 2007; Khodarahmi and Azadbakht, 2014). Some case–control studies have suggested increased risk of BC with increased fat intake (Thiebaut *et al.*, 2007), whereas this was not observed in most cohort studies (Kim *et al.*, 2006; Lof *et al.*, 2007) or pooled analyses (Smith-Warner *et al.*, 2001).

#### Saturated fat/fatty acids

The positive association between saturated fatty acids intake and BC risk has been suggested by several case–control and cohort studies, particularly in the etiology of hormone-sensitive rather than receptor-negative BC subtypes (Sieri *et al.*, 2014), and by a meta-analysis of 14 cohort studies (Khodarahmi and Azadbakht, 2014). However, a pooled analysis of eight cohort studies has shown a weak elevation of risk (relative risk=1.09) with replacement of saturated fatty acids intake by carbohydrate in an isocaloric diet (Smith-Warner *et al.*, 2001).

#### Monounsaturated fatty acids

**Fig. 1:**
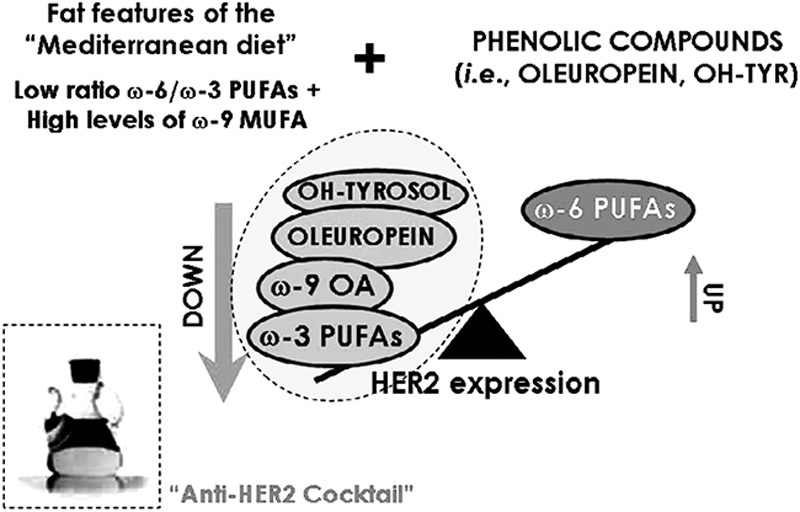
Olive oil as a naturally occurring ‘anti-human epidermal growth factor receptor 2 (*HER2*) cocktail’. In terms of protective effects against breast cancer, olive oil has a very favorable fat composition as it appears to combine a new composite biomarker for breast cancer, that is, a low *n*-6 : *n*-3 polyunsaturated fatty acid (PUFA) ratio, and elevated *n*-9 monounsaturated fatty acid (MUFA) levels (Menendez *et al.*, 2007).

An inverse association between monounsaturated fatty acids (MUFA) intake from extra-virgin olive oil and BC risk (Voorrips *et al.*, 2002), as well as its general protective effect (Pelucchi *et al.*, 2011), are attributable to MUFA’s innate oxidative stability, improvement of insulin resistance (Farnetti *et al.*, 2011), and to olive oil polyphenols – including hydroxytyrosol and oleuropein aglycone (Menendez *et al.*, 2007) (Fig. 1) – that have shown effective reduction of viability in various human BC cells lines (Owen *et al.*, 2004). However, MUFA intake from hydrogenated fat high in artificial *trans*-fatty acids (as in margarine) was linked to increased risk of BC (Kohlmeier, 1997).

#### *n*-3 polyunsaturated fatty acids

Systematic review of cohort studies and meta-analyses showed an inverse association between BC and *n*-3 PUFA and *n*-3 :  *n*-6 ratio, especially when confirmed in biological samples, such as adipose tissue, erythrocyte membranes, serum, and plasma (Rose, 1993; Simonsen *et al.*, 1998), possibly because of reduced inflammation, carcinogenic, and oxidative stress and enhanced insulin sensitivity (Larsson *et al.*, 2004), and recently shown reduced obesity trajectory (Simopoulos, 2016). In a cohort of women with early-stage BC, high eicosapentaenoic acid and docosahexaenoic acid intakes (>73 mg/day) from foods (marine sources) for 7.3 years reduced BC events by ∼25% and modulated BC risk biomarkers – both in premenopausal (Fabian *et al.*, 2015) and in postmenopausal (Hilakivi-Clarke *et al.*, 2002) women – suggesting their potential contribution toward BC prevention.

#### *n*-6 polyunsaturated fatty acids

**Fig. 2:**
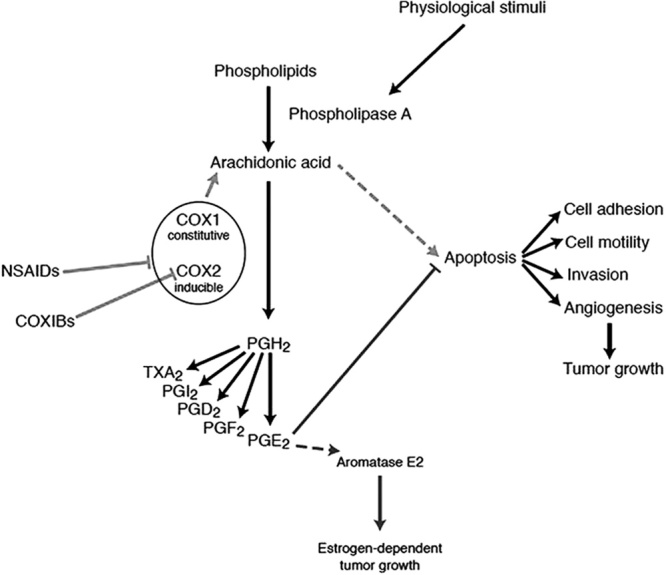
The mechanisms of action of aspirin (anticyclooxygenase products of arachidonic acid). Prostaglandin synthesis through arachidonic acid and the possible effects of cyclooxygenase inhibitors as chemopreventive agents of breast cancer by the intracellular accumulation of arachidonic acid, which directly promotes apoptosis and attenuation of positive feedback for proliferation and survival. COX, cyclooxygenase; COXIBs, COX2 selective inhibitors; PG, prostaglandin; TXA_2_, thromboxane A_2_ (Jacobo-Herrera *et al.*, 2014).

High intake of *n*-6 PUFA, primarily linoleic acid has been associated with a high prevalence of BC (Shapira, 2012). Increasing *n*-6 :  *n*-3 ratio – primarily of long-chain PUFA arachidonic acid (20 : 4) to *n*-3 (eicosapentaenoic acid 20 : 5) in plasma and adipose tissues – was associated with a proinflammatory response, altered adiponectin secretion, and development of MetS (Caspar-Bauguil *et al.*, 2012), enhanced cellular and DNA damage (Wirfalt *et al.*, 2002), and accelerated oxidative stress and proinflammatory effects. In contrast, aspirin, the inhibitor of cyclooxygenase 2 – the enzyme that converts long-chain PUFAs into their eicosanoids – was associated in a western high *n*-6 diet with reduced *n*-6 procarcinogenic/proinflammatory compounds (Fig. 2), resulting in improved BC survival and reduced BC and all-cause mortality, as well as with reduced relapse/metastasis when taken before diagnosis (Huang *et al.*, 2015) (although taking after diagnosis was not significantly effective) (Barron *et al.*, 2015).

**Fig. 3:**
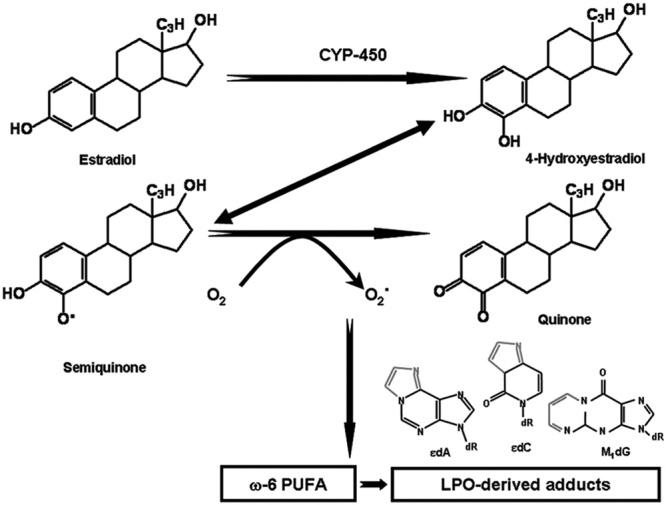
Proposed scheme for metabolic redox-cycling of 4-hydroxyestradiol leading to reactive oxygen species and lipid peroxidation (LPO) of ω-6 polyunsaturated fatty acid (PUFA); the resulting LPO byproducts such as 4-hydroxy-2-nonenal and malondialdehyde generate miscoding etheno-DNA adducts (edA and edC) and M1dG, respectively, that were analyzed in white blood cell of healthy female volunteers (Sun *et al.*, 2012).

A biochemical link between estradiol catabolism and *n*-6 PUFA, and resultant lipid oxidation-induced DNA damage (Fig. 3) was shown by in-vivo and in-vitro models (Sun *et al.*, 2012) through enhanced formation of miscoding etheno-DNA adducts (Bartsch *et al.*, 1999) in the white blood cells of women, but not of men (Nair *et al.*, 1997; Bartsch *et al.*, 1999) – with potential for cancer initiation and recurrence (Kiraly *et al.*, 2015) – indicating sex differences and women’s greater predisposition with a high *n*-6 diet, very common in the western diet.

The ‘Israeli Gender *n*-6 PUFA Nutrition Paradox’ hypothesis (Shapira, 2012) links Israeli women’s higher disease/cancer risk, relative to men’s, with women’s inherently greater desaturase activity and *n*-6 PUFA conversion into proinflammatory/oxidative/carcinogenic eicosanoids under conditions of a high *n*-6 diet – compared with men – and resultant worse international health ranking for women.

This further led to Israeli-Jewish women being first in the national cancer over heart disease mortality shift, and to decreasing the gender gap in all diseases and life expectancy, and subsequently to increasing Israeli-Arab women’s BC prevalence with *n*-6 consumption, gradually closing the gap towards Jewish women levels (Israel National Cancer Registry, 2008). This emphasizes the importance of sex-specific nutrition, especially versus BC.

#### Cholesterol

Cholesterol was suggested to accelerate and enhance tumor formation, aggression, and angiogenesis, whereas its blood levels are reduced during tumor development (Umetani and Shaul, 2011). A cholesterol metabolite – 27HC – may increase the proliferation of ER+ BC cells (Bjarnadottir *et al.*, 2013). The 27HC-producing enzyme, CYP27A1, which is expressed primarily in the liver and in macrophages, was significantly elevated within breast tumors, acting as an ER agonist and stimulating the growth and metastasis of tumors in several models of BC (McDonnell *et al.*, 2014b).

### Carbohydrates

#### Glycemic factors

A prospective cohort analysis (62 739 postmenopausal women, 1812 BC cases) showed no association between dietary carbohydrate or fiber intakes and overall BC risk, but rather an increased risk for BC with glycemic index (GI) among overweight women, and with increased carbohydrate intake, GI, and GL in women with high waist circumference (Lajous *et al.*, 2008). A recent prospective study (European Prospective Investigation into Cancer and Nutrition) reported increased BC risk associated with higher dietary GL, but not GI and total carbohydrate intake (Sieri *et al.*, 2013).

#### Refined sugars

Frequent consumption of SSB was associated with general and abdominal obesity (Hu and Malik, 2010), MetS (Barrio-Lopez *et al.*, 2013), fatty liver (nonalcoholic fatty liver disease) and T2DM (Malik *et al.*, 2010), and resultant pathometabolic/endocrine outcomes that are related to BC (Basaranoglu *et al.*, 2015). SSB may also reduce the age at menarche, whereas sugar-free (diet) soda and fruit juice consumption was not observed to affect it (Carwile *et al.*, 2015). Fructose – despite a moderate GI – has shown an association with increased lipogenic and proinflammatory effects, and nonalcoholic fatty liver disease (Alwahsh and Gebhardt, 2016). The association between refined sugar and increased risk of BC is further shown by enhanced mammographic breast density with higher intake (Duchaine *et al.*, 2014) and partially explained by its role in increased inflammation and induced 12-LOX signaling in BC development and metastasis (Jiang *et al.*, 2016).

#### Fiber

Fiber intake has been linked to a reduced risk of BC (by 5% for every additional 10 g/day), potentially by reducing the re-absorption of steroids in the bowel, especially soluble fiber with high absorption capacity, and further beneficial effects on insulin sensitivity (Aune *et al.*, 2012a). Through gut fermentation, grain fibers (especially from rye) reduce the toxicity of free bile acids and produce short-chain fatty acids such as butyrate, which yield anticancer effects, against BC. Fiber also enhances satiety and reduces the link between alcohol intake and BC risk (Chhim *et al.*, 2015). Lignans and phytic acid from legumes/pulses and whole grains have shown antioxidative and anticarcinogenic potential in general (Norhaizan *et al.*, 2011) and against BC in particular (Adlercreutz, 2010).

### Plants and phytonutrients

#### Vegetables

Total vegetable intake has been related inversely to BC risk, especially legumes/pulses and allium (Bao *et al.*, 2012) and vegetables, particularly cruciferous (Boggs *et al.*, 2010; Suzuki *et al.*, 2013) and raw vegetables (Turati *et al.*, 2015). Broccoli’s significant potential is because of the high content of sulforaphane, a potent inducer of detoxification enzymes such as NAD(P)H : quinone oxidoreductase 1 and glutathione-*S*-transferase. NAD(P)H : quinone oxidoreductase 1 and glutathione-*S*-transferase together prevent estrogen–quinone-mediated DNA damage and carcinogenesis (Yang *et al.*, 2013).

High intake of raw vegetables and olive oil showed protection against BC, specifically against *HER2*+ cancers (relative risk=0.25) as opposed to *HER2*− cancers (Sant *et al.*, 2007). Vegetables also reduce carbohydrate’s glycemic effect, suggesting their protection against BC-related risks of pathological IGF-1 and insulin metabolism (Imai *et al.*, 2014).

#### Fruits

Although no consistent association has been observed between total fruit intake and BC, high intake of specific fruits, such as citrus and rosaceae, has shown an inverse association (Bao *et al.*, 2012). Beyond their low ED and high nutritional density contributions to satiety, body weight management, and nutritional values, they reduced craving for sweets (Marcinkowski *et al.*, 2012). Fruit antioxidants – especially carotenoids and polyphenols – can reduce the oxidative stress following high GL, preferably eaten as whole rather than fruit juice (Peluso *et al.*, 2014).

#### Carotenoids

Women with higher circulating levels of α-carotene, β-carotene, lutein+zeaxanthin, lycopene, and total carotenoids may be at reduced risk of BC (Aune *et al.*, 2012b; Eliassen *et al.*, 2012), particularly among smokers and nonusers of dietary supplements (Larsson *et al.*, 2010). In BC cells, carotenoids inhibited IGF-1 induced growth, estrogen-induced proliferation, and further estrogenic activities (Hirsch *et al.*, 2007).

#### Polyphenols

Table olives are extremely rich in polyphenols (1–3% fresh pulp) (Charoenprasert and Mitchell, 2012) – mostly oleuropein and hydroxytyrosol – all having antioxidative effects, and some also exert antiproliferative effects against human BC cells (Cicerale *et al.*, 2012; Parkinson and Keast, 2014). Peach and plum polyphenols (Vizzotto *et al.*, 2014) have also been shown to reduce BC cell viability and inhibit their proliferation (Vizzotto *et al.*, 2014), and coffee was associated negatively with BC risk of overall and ER+/PR− (Oh *et al.*, 2015). Among polyphenols, carnosic acid, curcumin, silibinin, sulforaphane (Veprik *et al.*, 2012), and punicalagins (from pomegranate) (Shirode *et al.*, 2015) showed reversal of epigenetic alterations and carcinogenesis, including initiation, promotion, and progression (Pan *et al.*, 2015), and some, for example, green tea catechins, showed synergy with certain conventional anti-BC chemotherapy agents such as tamoxifen or raloxifene (Yiannakopoulou, 2014).

#### Soy isoflavones

Soy isoflavone intake was shown to reduce the risk of BC in both premenopausal and postmenopausal women in Asian countries, although no association was observed for women in western countries for either menopausal status (Chen *et al.*, 2014). Soy isoflavones were also associated with a lower risk of recurrence among postmenopausal patients with BC and individuals receiving endocrine therapy (Guha *et al.*, 2009; Kang *et al.*, 2010), for example, tamoxifen (Guha *et al.*, 2009). A recent meta-analysis summarizing 14 studies further showed that patients who consume moderate amounts of soy throughout their life have a lower BC risk (Dong and Qin, 2011). However , several interventional studies using high doses of soy estrogens have shown changes in breast nipple fluid that would predict higher rates of BC (Willett, 2003). Correspondingly, experts recommend adhering to a moderate intake of isoflavones rather than using their sources as protective foods.

### Food processing

Food processing has a significant impact on the total diet effect. Some nutrients may be leached out, whereas others – such as carrot and pepper carotenoids – become more available when cooked in oil, especially olive oil, which increases bioavailability and is itself rich in anti-BC compounds (Agnoli *et al.*, 2015). Conversely, some antioxidants can become pro-oxidants when processed at high temperatures, open heating, grilling, and/or frying. Red meat is especially sensitive to processing, with increasing risk of BC with well-done versus medium rare and low intake among postmenopausal women (Fu *et al.*, 2011).

### Summary and conclusion

The present paper presents the multivariate nature of diet association with BC. The most updated support of the potential for BC prevention comes from previous understanding of the endometabolic trajectories under an obesogenic environment, and recent studies showing encouraging results confirming decreased risk across genetic types and menopausal status, through adherence to six to eight basic recommendations, compared with nonadherence. The dietary recommendations include low-ED, low-GL, and nutritious plant-based foods, with minimal intake of animal foods, particularly red/processed meat and alcohol; other lifestyle recommendations include management of physical activity, body/abdominal fatness and adult weight gain, and extended breastfeeding duration.

Nutritional strategies include Mediterranean, DASH, and/or Okinawan patterns, which were found to be more easily applied than the recommendations based on dietary analyses and composition, are especially important during critical risk periods.

Taken together, the existing science supports the potential for lifelong BC prevention, starting from the earliest critical period – *in utero* – throughout the life cycle, with a nutritional approach aiming both for primary prevention of carcinogenesis as well as modification of the metabolic trajectory against disease occurrence and recurrence – to improve survival and quality of life. Increasing incidence of BC, already beyond one out of every eight women, highly necessitates support of the population by health authorities for lifelong BC prevention.
